# Behavioral and Dietary Strategies for Weight Loss and Weight Loss Maintenance Among Black/African American Adults and the Potential Role of Media: A Narrative Review

**DOI:** 10.3390/nu17040617

**Published:** 2025-02-08

**Authors:** Enid A. Keseko, Alexis Bell, Gabrielle M. Turner-McGrievy

**Affiliations:** Department of Health Promotion, Education, and Behavior, University of South Carolina, Columbia, SC 29208, USA; ekeseko@email.sc.edu (E.A.K.); ajb35@email.sc.edu (A.B.)

**Keywords:** nutrition and diet, behavioral, weight loss, media, African American

## Abstract

Background: Research shows that media-based dietary and behavioral strategies can aid weight loss, but limited studies have been conducted among Black/African American adults. Objective: This review examines the literature on dietary and behavioral strategies for weight loss and maintenance among Black/African American adults, identifying the types of media used alongside these strategies. Methods: The PubMed, Web of Science, CINAHL, and Communication & Mass Media databases were searched for peer-reviewed articles with no restrictions on the publication date. Two reviewers conducted the screening. Studies were included if they had >75% Black/African American adult participants (18 years and older), included behavioral or dietary strategies, had a media component, weight loss or maintenance as an outcome, and published in English language. Results: Nine studies (randomized control trials (n = 5), mixed-method studies (n = 2) and qualitative studies (n = 2)) were included. Behavioral strategies used to lose and maintain weight included goal setting (n = 4), self-monitoring (n = 5), and weekly self-weighing (n = 2). Limiting sugary drinks (n = 3), limiting junk and high fat foods (n = 5), aiming for a set number of calories per day (n = 3), portion control (n = 4), and increasing fruits and vegetable intake (n = 3) were the most common dietary strategies used to lose and maintain weight. Media used in the intervention studies included mHealth text messaging (n = 2), Facebook (n = 2), a website (n = 1), television (n = 1) and a mobile app (n = 1). Conclusions: The findings highlight the limited research on the utilization of media for behavioral and dietary weight loss strategies among Black/African American adults, indicating a need for future studies to explore and optimize media-based strategies for this population.

## 1. Introduction

The obesity rate among adults in the United States has increased significantly in recent years [1,2], with non-Hispanic Black adults having the highest age-adjusted prevalence [3,4,5]. This alarming trend highlights the need to pay urgent attention to the prevalence of obesity, as it may be more common than current studies suggest [6]. Recent research has identified several effective strategies used in obesity interventions for weight loss and maintenance including individualized dietary strategies such as energy deficit, low-calorie diets, and meal timing [7]. Behavioral strategies such as having healthy foods at home, regular breakfast intake, increasing vegetable consumption, as well as increased physical activity or regular exercise, are other potential weight loss and weight loss maintenance strategies [8]. Other diet-related and behavioral strategies include self-monitoring, problem solving, social support, goal setting, and shaping knowledge [9,10]. A review of a 25-year research program on behavioral weight loss and weight loss maintenance showed that changes in self-regulation, self-efficacy, and mood were the most salient predictors of change in dietary intake [11]. Although limited [12], culturally appropriate weight-loss management interventions among Black/African American adults have been conducted with the use of strategies such as nutrition education and physical activity [13,14]; extended care after obesity treatment [15]; and support from community health workers [16].

Media outlets such as radio, television, print media and digital media including social media and websites play key roles in supporting health [17]. A systematic review with meta-analyses of 51 articles showed that web-based interventions on weight change in adults with overweight and obesity were effective [18]. Strategies used in these interventions included self-monitoring for weight and behavior change, goal setting for weight, and social support [18]. In other studies, web-based digital health interventions have led to greater short-term weight loss than offline interventions in adults with overweight and obesity [19]. Additionally, behavioral weight management interventions using mobile devices (mHealth) for self-monitoring have led to moderate weight loss and higher adherence [20]. Social media-delivered interventions also capitalize on the widespread use of social networking platforms such as Facebook to promote weight loss strategies and provide social support [21] despite evidence of inadequately controlled and powered pilot studies [22].

Mobile apps have been used to facilitate dietary self-monitoring and self-regulation for weight loss. App users have been more consistent in tracking dietary intake compared to traditional methods, and frequent recording of weight [23,24]. Moreover, app usage for goal setting and self-regulation towards diet has shown potential for clinically significant weight loss [25]. Apps with evidence-based content have been shown to support behavior change, healthy eating habits and successful weight loss if conducted effectively [26]. Remote weight-loss programs combining mobile applications with daily self-weighing, and calorie restriction have also been found to have positive results [27]. Mobile phone apps have also been used in conjunction with text messaging [28]. Text messaging has emerged as a valuable tool in dietary and behavioral weight loss strategies, as evidenced by significant weight loss and maintenance through text messaging interventions [29,30].

Although fewer recent studies have focused on mass media interventions, older research suggests the effectiveness of mass media interventions in promoting dietary weight loss and behavior change. Television-delivered interventions [31] and documentaries [32] have shown promise in this regard. Avatar-based weight loss interventions assessing empirical support were found to be effective in the short and medium term and improved in the long term (12 months) [33]. These studies collectively suggest that media-based interventions, delivering behavioral and dietary strategies, can be effective for weight loss.

Obesity and its associated chronic diseases such as diabetes, hypertension, and cardiovascular diseases, are disproportionately prevalent among Black/African American adults than in the general US population [5,33]. These health disparities arise from socio-economic factors, limited access to healthcare, environmental influences, and systemic inequities that affect health outcomes, contributing to higher rates of morbidity and mortality in this population [5,34,35]. Research indicates that weight management strategies tailored to the cultural and contextual needs of Black/African American adults can be more effective than general recommendations by capturing dietary preferences and tailored engagement [5,36]. Generally, weight loss and weight loss maintenance interventions that utilize media in the delivery of dietary and behavioral strategies have shown promise [37,38,39], but few of these studies have been conducted among Black/African American populations [12]. Given the challenges faced by this population and the potential for media to reach and engage diverse audiences, it is critical to explore how media can be leveraged to address obesity in culturally relevant ways. The goal of this review is to examine behavioral and dietary strategies for weight loss and weight loss maintenance used among Black/African American adults and identify the types of media (i.e., digital media, mass media, etc.) used alongside the strategies. Through this review, we hope to advance the understanding of media-based interventions and their potential to improve weight management outcomes among Black/African American adults.

## 2. Methods

### 2.1. Study Eligibility Criteria

The inclusion criteria for studies were the following: (1) studies that included at least 75% Black/African American adults (18 years and older); (2) studies implementing behavioral or dietary strategies; (3) studies with at least one media component; (4) studies with weight loss or weight loss maintenance as an outcome; and (5) studies published in English language.

### 2.2. Search and Selection Process

Four scientific research databases (PubMed, Web of Science, CINAHL, and Communication and Mass Media), were electronically searched for peer-reviewed articles, with no restrictions on the publication date. An initial test search was performed using behavioral strategies or dietary strategies in combination with weight loss, weight loss maintenance, African American and media. Synonyms related to the search terms were added to the search strategy (Table 1). Mesh terms for behavioral strategies, dietary strategies, weight loss maintenance, African American and media were searched in combination with Boolean operators AND, OR. The selection of peer-reviewed research studies was achieved by screening abstracts using the study criteria by two reviewers, ensuring reliability and consistency in the selection process and minimizing bias. Studies were screened by title and abstract, followed by full text to determine eligibility.

## 3. Results

**Figure 1:**
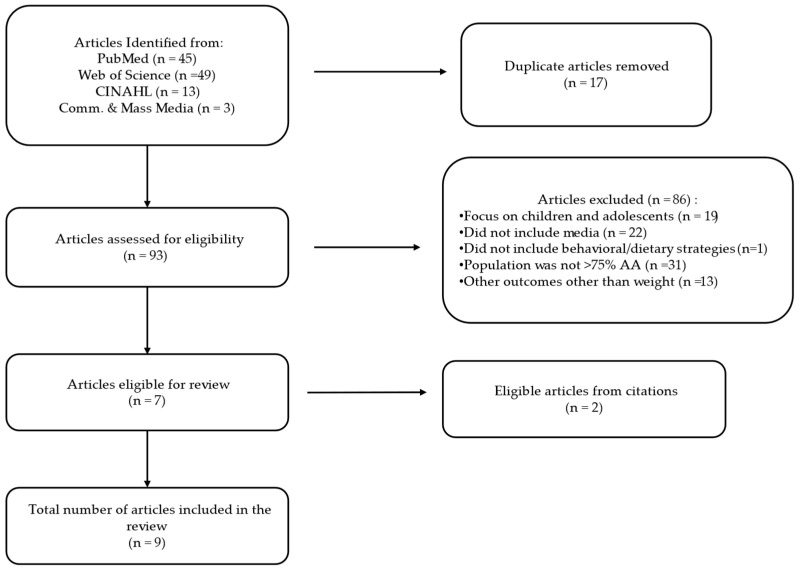
Study selection.

There were 110 studies identified from the electronic database search (Figure 1). Duplicates (n = 17) and studies that did not meet the inclusion criteria (n = 86) were removed, resulting in seven studies that met the eligibility criteria. A further search was performed on the reference lists of eligible articles, and two additional studies were identified. A total of nine articles were therefore included in this review (Figure 1). Data related to the purpose of this review were extracted from each of the eligible studies as illustrated in Table 2; author, purpose, study design and theoretical framework, population and sample characteristics, behavioral and dietary strategies, type of media (used either as a part of the intervention components or as the main component of the intervention), main outcomes and results.

### 3.1. Characteristics of Sample Studies

Studies were conducted in the United States [40,41,42,43,44,45,46,47] or Europe [48]. Overall, the studies encompassed various aspects of utilizing digital platforms and interventions to address weight management among Black/African American adults. The three main purposes of the studies were to facilitate postpartum weight loss [40,42], enhance weight loss among adults with overweight or obesity [41,43,44], and to explore perceptions on overweight and obesity among African American women and examine communication preferences [45,46,47]. The randomized control trial [40,41,42,43,44] was the most common design used to address these purposes, followed by mixed methods [45,46] and qualitative design [47,48]. Most of the studies [40,42,46,47,48] did not include a description of a theoretical framework. One study included multiple theoretical frameworks (i.e., Health Belief Model (HBM), Transtheoretical Model (TTM) and Self-Regulation theory) [43], whereas the other studies included the Social Cognitive Theory (SCT) [41,45] and Social Action Theory [44]. The eligibility criteria for study participants often encompassed age, race (primarily African American or Black), interest in weight loss, smartphone ownership, and absence of cognitive issues. Eight studies included women only in their study sample [40,41,42,44,45,46,47,48]. Sample sizes ranged from as low as 18 participants in a feasibility pilot study [40] to 413 participants in a mixed-method study [46]. Participants ranged from 18 to 65 years in age across all studies, while the study durations varied from 3 months to 6 months with varying lengths of follow-up. Overweight or obesity, defined as a BMI of 25 kg/m^2^ or higher, was prevalent among participants in all studies. The studies had an all-African American population [40,41,42,43,44,45,46,47], at least 75% African American [40] or a population of African descent [48].

### 3.2. Synthesis of Findings

Based on the study purpose, common themes from the retrieved data have been described following the study designs. Some studies used a combination of both behavioral and dietary strategies while other studies used a combination of media platforms.

#### 3.2.1. RCTs

The primary outcome for all the intervention studies was weight loss. None of the studies assessed weight loss maintenance post intervention. Among the RCT studies included in this review, goal setting [40,41,42,43], self-monitoring or management [40,42,43,44], and weekly self-weighing [40,42] were the most common behavioral strategies. Other behavioral strategies that were used alone or in combination with the above included walking 30 min or 5000 steps/day [40,43,44], obtaining 7 h of sleep/day [40], recognizing, and modifying barriers [41].

Dietary strategies included limiting sugary drinks [40,41,43], limiting junk and high fat foods [40,41,42,43,44], calorie targeting [40,42], portion control [41,43,44], increasing fruits and vegetable intake [41,42,44], reading food labels [41], and meal timing [42,43]. Behavioral strategies were used in combination with some of the dietary strategies, such as goal setting around each dietary strategy [40,43].

The types of media used in the studies included mHealth text messaging [40,43], Facebook [40,42], eHealth using a website [41] a mobile app [42], and television [44]. Text messaging and social media integration, such as private Facebook groups, were used to enhance social support aspects and deliver intervention content. Text messaging [40,43] and in-app messaging [42] were used for strategies such as goal setting and self-monitoring. Intervention participants in three of the five studies [40,43,44] experienced greater body weight loss than control participants.

#### 3.2.2. Qualitative Studies

Qualitative studies aimed to understand perceptions and preferences regarding digital weight loss interventions among women through focus groups. In a formative study [47], when participants were asked about their social media use for health and nutrition, they reported that they mostly engaged with Facebook more than Instagram and Twitter for information related to dietary strategies for weight loss. Participants also indicated a preference for mHealth interventions that integrated social media or text messaging to provide social support. In addition, interventions that used a mobile app for self-monitoring of dietary intake were preferred among the participants. However, participants expressed concern that most existing apps did not have ethnic foods for Black people. In the second study [48], participants sought dietary advice from dietitians, but did not specify the strategies they were advised to use for weight loss. Participants also sourced weight- and diet-related information from internet searches and watching television. Participants reported skepticism towards leaflets or print media as sources of diet-related information and indicated that television programs influenced their weight loss perceptions. In both studies, participants preferred media that included behavioral strategies for weight loss over providing information on diet or dieting alone.

#### 3.2.3. Mixed-Method Studies

The mixed-method studies explored various perspectives on weight management among African American women combining qualitative and quantitative approaches for an in-depth investigation. Using SCT as an interview framework, one mixed-method study investigated perceived weight loss obstacles among low-income African American women [45]. In-depth interviews were performed to provide responses for open-ended questions while closed-ended survey questions were read and recorded by the interviewer. Findings revealed that participants primarily accessed health information through television and radio, while the internet and magazines were less commonly used. Challenges related to low self-efficacy were prominent, with women reporting difficulty in avoiding sweets and fatty foods, adhering to a diet plan, and lacking a supportive network. Self-monitoring of diet and exercise was also infrequent among participants. The second study examined the link between health literacy and sources of diet information among African American women [46]. Quantitative data were collected via surveys over 6 months while a sample of women who completed the surveys participated in focus groups. Qualitative data confirmed the quantitative data showing that television was the most frequently used dieting information source followed by women’s magazines. Women with higher health literacy levels were more likely to use the internet for diet information and tended to focus on specific aspects of weight-loss programs, such as toning, rather than simply reducing body weight. Behavioral and dietary strategies for weight loss that emerged from the focus groups include exercising regularly, avoiding fast foods, reducing fat and sugar intake, using smaller plates for portion control, and eating salads. In terms of information on weight management strategies, participants identified portion control, calorie recommendations, stress management, and self-affirmation as essential.

## 4. Discussion

This narrative review aimed to describe behavioral and dietary strategies used among Black/African American adults for weight loss and weight loss maintenance, as well as how media has been used in these studies. The review examined various behavioral and dietary strategies in weight loss studies that had a media component in nine studies. Of the nine studies, eight studies included women only in their study sample, of which two focused on postpartum weight loss. Few weight loss studies among Black/African American adults have focused specifically on men [36] and most of the studies including men have recruited more women than men [49]. This highlights a need for more inclusive research that engages both Black/African American men and women in weight loss efforts. Ensuring that the studies are culturally and gender sensitive, involving gate keepers and maintaining transparency are some strategies that can help increase participation of African American men in health research [50].

Culturally tailored weight loss programs are essential for effectively addressing obesity in diverse populations [36]. In this review, one intervention study benefited from extensive formative research with the target group which ensured that the program aligned with cultural values and preferences [44]. Cultural, social, and psychological factors are crucial for understanding eating habits and body image dissatisfaction [51]. The findings from the qualitative studies showed that integrating holistic components in weight loss programs such as emotional eating and body dissatisfaction can be beneficial [47]. A key concern voiced by participants was the lack of ethnic food options in many existing weight management apps, highlighting the importance of including culturally specific dietary choices in digital interventions [47]. This gap underscores the need for developers to create platforms that reflect the diverse culinary traditions of their user base, thus enhancing the app’s relevance and usability. Additionally, tailoring communication strategies based on ethnic and cultural differences can optimize engagement [47].

None of the intervention studies examined weight loss maintenance as an outcome yet Black/African American adults generally lose less weight and maintain a lower percentage of weight loss compared to their counterparts [52,53]. Digital technologies show promise in supporting weight loss maintenance, which is often more challenging than initial weight loss [54]. Several studies have found that eHealth interventions using text messaging, email, and web-based systems can be effective for short-term weight loss maintenance, typically 3–24 months [55]. Considering the evidence, it is essential to explore digital platforms to enhance weight loss maintenance among Black/African American adults.

Behavioral and dietary strategies identified in the studies in this review such as regular meal rhythm, having breakfast, reducing dietary fat, self-monitoring, and having a physical active lifestyle, have been associated with weight loss and maintenance [56]. One similarity in RCT studies with greater weight loss among the intervention group [40,43] was the use of more than at least six behavioral dietary strategies and study lengths of more than 12 weeks. This finding suggests that multifaceted approaches in weight loss interventions among Black/African American adults that extend for longer periods are effective. However, findings from one qualitative study also showed that participants preferred short programs of 4–6 weeks due to their perceived attainability [47]. A younger participant indicated that a short program could provide a solid foundation for understanding healthier eating and exercise routines [47]. Several participants found this duration “doable” for weekly check-ins and lifestyle tracking. Middle-aged and older participants also saw shorter programs as beneficial for a “jump-start” or as a refresher, with comments highlighting that a shorter duration felt manageable and less likely to interfere with everyday commitments. Nevertheless, participants also recognized that longer interventions (6 to 12 months) could yield better outcomes for weight loss and maintenance [47].

The preference of shorter studies could explain why Black/African American adults may struggle with adherence to long behavioral weight loss or weight loss maintenance programs [57]. One approach to balance these varied needs is implementing personalized intervention components. Personalized interventions have been shown to respond to individual progress and help maintain engagement over time [58]. This approach may help bridge the gap between preference for shorter programs and the necessity of sustained support for effective weight management. Another potential solution could be to design phased or time-based interventions that start with an intensive 4- to 6-week program to build momentum, followed by additional support over several months, an approach that has been beneficial for improving long-term weight loss [59]. Lastly, incentives such as financial rewards and flexible participation were seen as crucial for maintaining engagement and commitment from one qualitative study [47], aligning with findings that monetary incentives can increase participation in weight loss programs [60]. These findings highlight the importance of holistic planning when designing weight loss interventions for African American adults, regardless of program duration.

While various media platforms have had negative influence on overweight and obesity including weight bias and weight stigma [61], findings support that the use of media, such as social media in weight management efforts, is a growing field [62,63]. Media channels used in the studies in this review included text messaging, social media such as Facebook, websites, and mobile apps. Facebook emerged as the most frequently used social media platform for engagement and social support, a finding consistent with other studies on weight loss in adult populations [22,64]. The popularity of Facebook can be attributed to its private group capabilities, which foster community engagement [64]. In the RCTs examined within this review, participants in the intervention group who received social support and had increased engagement tended to achieve more weight loss. Interventions that have higher social support have been found to have higher adherence and adherence is necessary for initial and long-term weight loss [65]. Social support has also been identified as a critical factor in enhancing behavioral strategies such as perceived control and self-efficacy for weight management among African American women [66]. In health promotion, social support often focuses on structural support yet functional support, such as perceived support, correlates more strongly with health outcomes [67,68]. Moreover, social media use has been shown to enhance perceived social support [69]. Notably, intervention groups that experienced greater weight loss often utilized Facebook for social support and self-monitoring [40]. Online health communities provide social support, which is crucial for successful weight loss and behavioral change, potentially leading to increased self-efficacy and actual weight loss [70].

Previous studies have documented weight loss attempts using various media platforms including newspaper and magazines [71] or books and magazines for post-partum weight loss [72], YouTube [73,74] and TikTok videos [75,76]. Additionally, previous studies have demonstrated the effectiveness of books [77] and documentaries [32,78] in promoting weight loss and maintenance across various demographic groups. Findings from the current review show the role of traditional (e.g., print and broadcast media) and digital media (e.g., web-based, social media, and mobile apps) in weight management. Only one RCT study utilized television and supplemental print material to deliver an intervention [44], yet findings from the mixed-method studies [45,46] and one qualitative study [48] highlight television and women magazines as primary sources of health information. While some studies in this review included media such as Facebook, websites, mobile apps, and television, there remains a need to explore the potential impact of other media channels including print media (e.g., books and magazines), audio-visual media (e.g., documentaries), and video sharing platforms (YouTube, TikTok) in promoting weight loss and weight loss maintenance studies among Black/African American adults.

In this review, no single media platform consistently outperformed others in the intervention studies. Intervention studies demonstrated mixed outcomes for media strategies used in weight management. The use of interactive digital platforms, such as mobile apps, tailored text messaging, and social media, aligns with findings that show use of digital media for delivery of interactive content to support behavior change in weight loss programs [79]. Social media platforms like Facebook facilitated virtual support networks to promote engagement and self-monitoring, but success often depended on participants’ consistent involvement [40,42], echoing earlier studies showing that long-term adherence is challenging, with engagement generally declining over time [80]. The mixed outcomes of traditional media strategies, such as television programs, align with prior research that has shown the effectiveness of interactive health messages to depend on various factors, including message content and individual characteristics [81]. While a television-based intervention reported short-term successes [44], findings from the qualitative studies [48] highlight the need for credibility and realism in traditional media to avoid skepticism, which has also been noted in past reviews of health communication strategies [82]. These findings underscore the importance of incorporating media strategies that are not only interactive and culturally tailored but also credible to enhance both engagement and long-term adherence.

Understanding the theoretical basis of interventions for weight loss and weight loss maintenance is essential for maximizing their effectiveness [83]. Among the studies reviewed, three theory-based interventions demonstrated improved outcomes through targeted approaches to behavior change. In one intervention, participants benefited from theory-informed modules (Dietary Approaches to Stop Hypertension (DASH) education modules) which entailed self-management/regulation, motivational coaching to enhance social support, goal setting, and reciprocal determinism [41]. In another intervention, participants benefited from structured messaging aimed to improve their readiness to change and maintenance of change by constructing messages based on theory for content focused on cognitive (e.g., motivation level), behavioral (e.g., self-monitoring) and emotion aspects for strategies such as increasing exercise, portion control, reducing fat and sugar intake, having more balanced diet and eating when hungry [43]. In the third intervention, participants were empowered to take actionable steps towards change by observing social workers who modeled effective problem-solving techniques, in addition to empowerment to adopt self-monitoring and regulating personal behavior (e.g., low-fat cooking, portion control, active lifestyle choices) in alignment with social goals [44]. The constructs emphasized in these interventions—such as self-regulation and reciprocal determinism from SCT, stage progression from TTM and social interaction from Social Action Theory—closely align with the core principles of each theoretical model, demonstrating how these frameworks support targeted behavioral change in weight management.

Findings from a mixed-method study [45] revealed critical barriers to weight management including low self-efficacy, low self-esteem, lack of support, and limited self-monitoring, which align with SCT. These findings highlight the importance of grounding weight loss and maintenance interventions in theory, as public health interventions with a theoretical base are more effective than those without [84]. Although some of the intervention studies in this review included theory-based frameworks, none reported changes or improvements in the specific constructs they were based on e.g., self-regulation and problem solving. Future research could benefit from measuring these constructs directly to better understand how these frameworks vary in their impact.

Research suggests a complex relationship between stress, eating behaviors, and weight among African American adults, particularly women. Participants in one mixed-method study identified stress management as a key area of need, because of stress-related eating [46]. While stress exposure is generally associated with unhealthy eating patterns across racial/ethnic groups, evidence is mixed regarding differences in susceptibility [85]. Chronic stress and negative emotions like anger and guilt are associated with emotional eating and higher BMI [86]. Interventions that combine stress management strategies with behavioral weight control have shown promise for African American women with moderate to high stress levels [87]. Only two RCTs [41,44] in this review incorporated stress management as a strategy but did not report on any stress measures in their outcomes, highlighting the need for future research to explore its value within weight management interventions for Black/African American adults.

A notable limitation of this review is the concentration on studies involving Black/African American women, including pregnant women, which may affect the generalizability of our findings to the larger Black/African American community, including men and non-pregnant women. While this emphasis offers valuable insights into the unique experiences of women, future research should incorporate more male participants to ensure the applicability of weight management strategies across the broader population. Additionally, one of the included studies reported that 78% of participants were non-Hispanic Black, representing the majority of the sample, but did not provide separate data for this subgroup [40]. While the inclusion criteria ensured that this review predominantly involved Black/African American participants, the lack of disaggregated data presents a challenge in drawing population-specific conclusions. Future studies should prioritize reporting disaggregated data for diverse participants to enhance the clarity and relevance of results.

## 5. Conclusions

The integration of findings from intervention, mixed-method and qualitative studies highlights the importance of comprehensive behavioral and dietary approaches to weight loss and maintenance studies for Black/African American adults. This review highlights the limited research on the use of media in nutrition interventions targeting Black/African American adults, with only nine studies meeting the inclusion criteria. Given the extensive influence of media in daily life, this gap underscores an opportunity to leverage media for promoting healthier diets and weight loss in this population. To maximize the potential of media in supporting dietary behavior change, future research should expand to include a diverse representation of Black/African American men and focus on developing theory-grounded weight loss and weight loss maintenance interventions.

## Figures and Tables

**Table 1:** Key word terms used within electronic database searches.

Dietary Strategies	Behavioral Strategies	Weight Loss	African American	Media
Diet strategies	Behavior strategies	Weight loss maintenance	Black American	Social media
Diet intervention	Behavior intervention	Weight management	African American adult	Television
Healthy eating intervention	Behavioral strategy		Ethnic minority	Radio
Diet	Behavioral intervention		Black	Newspaper
Dietary	Health strategies		Afro-American	Magazine
Nutrition	Health behavior			Film
Nutrition strategy	Behavior change			Documentary
Nutritional strategies				Broadcast media
Nutrition intervention				Audiovisual media
Mindful eating				Web-based
Dietary changes				

**Table 2:** Synthesis of studies involving weight loss and maintenance behavioral and dietary strategies and media approaches.

Author	Study Purpose	Study Design, Theoretical Framework	Sample Characteristics	Behavioral, Dietary Strategies	Role of Media	Key Results
Intervention Studies						
Herring et al., 2014 [40]	Examine the feasibility, acceptability, and initial efficacy of a technology-based weight control intervention.	14-week randomized controlled trial; No theoretical framework.	N = 18, 78% non-Hispanic Black and 22% Hispanic; mothers aged ≥ 18 years; BMI ≥ 25 kg/m^2^; newborn delivered within past 2 weeks to 12 months; enrollment weight surpasses pregnancy weight by 5 kg.	Limit sugary drinks; Limit junk and high fat foods to no more than one per day; 1200–1500 cal per day; Walk 30 min or 5000 steps each day; 7 h of sleep per day weekly self-weigh; Personal goal setting was implemented for each strategy.	Daily text messages and self-monitoring prompts via text. Skills training and virtual support via Facebook posts.	Intervention group participants ate less food and lost more baseline body weight compared to the control group.
Staffileno et al., 2018 [41]	Promote a healthy lifestyle through increased physical activity and improved nutrition.	12-week feasibility randomized trial, pre-post design; Social Cognitive Theory.	N = 35; African American women, aged 18 to 45 years; untreated prehypertension (120–139 mm Hg and/or 80–89 mm Hg); BMI > 35.	Behavioral: Goal setting; recognize and modify barriers; Maintain changes; Prevent relapse. Dietary (Based on DASH): Setting dietary goals; Reading food labels; Incorporating non-fat or low-fat dairy; Reducing saturated/total fats; Increasing fruits/vegetables intake; Portion control; Whole grains, nuts, and fiber inclusion.	Intervention (i.e., online modules) was delivered through a website customized for the participant.	Changes in clinical outcomes (systolic BP, diastolic BP, weight, and BMI) did not differ across treatment groups. DASH group participants successfully modified their diet, resulting in improved total DASH scores and significant effects across various components.
Napolitano et al., 2021 [42]	Examine the feasibility of a digital healthy body weight intervention.	12-week feasibility randomized trial; no theoretical framework.	N = 136; African American/Black women aged 18–40 years; BMI 25–40; postpartum 3 days after giving birth.	Adapted from Diabetes Prevention Program: Limit sugary drinks like juice and soda to no more than 1 per day; Limit junk and high fat foods to no more than 1 per day; Cover one-half of the plate with vegetables at lunch and dinner; Regular meal pattern, eat a meal or a healthy snack every 4 h; 1200 to 1500 calories per day; No late-night meals or snacks; Weekly self-weighing.	Mobile app integrated with a private Facebook group whereby Facebook posts were used to create community and address neighborhood and social environment factors e.g., how to eat out. In app messaging also entailed goal setting and self-monitoring.	Approximately half of participants accessed the app and set a goal ≥ one time, but <10% reported achieving a nutrition or activity goal. Both groups did lose significant weight from baseline.
Lin et al., 2015 [43]	Investigate whether a behavioral theory-based mHealth intervention would enhance weight loss.	6-month randomized control; HBM; TTM and self-regulation theory.	N = 124; African American adults aged 21+ years; BMI > 27.	Increasing movement; Increasing exercise; Portion control; Balancing the diet; Reducing fat intake; Reducing sugar intake; Eating when hungry.	Mobile health intervention. entailed tailored rapid interactive mobile messaging. Messages addressed self-monitoring, cues to action; reminders; motivational level.	Mean weight loss at 3 months was greater in the intervention group compared with standard care group at 6 months.
Risica et al., 2013 [44]	Develop and evaluate a culturally tailored weight control cable TV program.	12 week randomized 5 group design with follow-up; Social Action Theory.	N = 363; 84% African American/Black women; BMI > 22.	Decreasing fat; Decreasing portion sizes; Increasing physical activity; Decreasing sedentary behavior; Increasing fruits and vegetables; Stress reduction; Self-management.	Participants watched a weight control program on TV.	The TV intervention group was associated with decreases in BMI and dietary fat and increased physical activity at 3 months and differences remained significant at 8- and 12-month follow-up for dietary fat. Over half of participants read print materials. Reading more written materials was associated with lower dietary fat at 3 months, which did not persist to the 12-month follow-up.
Mixed-Method Studies						
Mastin et al., 2012 [45]	Investigate perspectives on overweight and obesity using SCT as an interview framework.	Mixed-method: closed-ended surveys and open-ended interviews; SCT.	N = 46; African American women; age 18–65 years; BMI 25.8–70.9.	Participants rarely had habits on the following: Diet and exercise; Self-monitoring; Goal setting; Diet planning; Label reading.	Participants mostly relied on television and radio for their health information. The internet and magazines were used less often.	Participants indicated to have low self-efficacy: e.g., inability to avoid sweets and fatty foods, stick to a plan and lack of a support group as obstacles to losing or maintaining weight. Participants rarely self-monitored for diet and exercise.
James et al., 2015 [46]	Examine link between health literacy and sources of dieting information, the weight-loss methods used, and the information needed to manage weight.	Mixed-method; surveys and 7 focus groups; no theoretical framework.	N = 413; African American women; age mean 35.6; focus group sample N = 50.	Strategies needed to manage weight were the following: Portion control; Calorie recommendations; Stress management; Increasing self-esteem; Affirmation.	Sources of dieting information were the following: Television (48%); Women’s magazines (37%); Internet (31%); Diet books (27%); Newspaper (16%).	Participants with adequate health literacy appeared more focused on the details of the reality weight-loss programs instead of losing weight. Participants with adequate health literacy were more likely to use the internet to find dieting information.
Qualitative Studies						
James et al., 2022 [47]	Explore ideal components to consider in developing a mHealth weight management intervention.	12 focus groups; no theoretical framework.	N = 36; African American women aged 18–65; BMI ≥ 25 kg/m^2^.	Strategies identified in the themes: Holistic program that goes beyond dieting; Social media integration for support and sense of community; Self-monitoring app; 2-way text messaging; and programs of varying lengths; Meaningful incentives.	Participants regularly engaged with the following: Facebook (85%); Instagram (44%); Twitter (30%); Most (70%) searched online for dieting information within the past 12 months.	Several participants expressed reservations on programs that focused strictly on dieting. Several participants in all age groups had a health/nutrition app on their smartphones. Facebook was mentioned in all age groups. Short programs of 4 to 6 weeks were the most appealing.
Hartman et al., 2015 [48]	Explore similarities and differences in the use and perception of communication channels to access weight-related health promotion.	8 focus groups; no theoretical framework.	N = 48; women of Ghanaian, Antillean/Aruban, or Afro-Surinamese background living in Amsterdam; BMI ≥ 25 kg/m^2^.	Some women had used dietary advice from a dietitian but no details on dietary strategies.	Sources of weight-related information: Information leaflets; Searching the Internet; Watching television.	Leaflets were often thrown away. Television programs promoting extreme weight loss in Antilleans and Surinamese reinforced their need for quick and extensive weight loss, while Surinamese women did not find these messages credible. Documentaries were a source of weight loss information.

## Data Availability

No new data were created or analyzed in this study. Data sharing is not applicable to this article.
